# Disrupted cortical brain network in post-traumatic stress disorder patients: a resting-state electroencephalographic study

**DOI:** 10.1038/tp.2017.200

**Published:** 2017-09-12

**Authors:** M Shim, C-H Im, S-H Lee

**Affiliations:** 1 Department of Biomedical Engineering, Hanyang University, Seoul, Korea; 2 Clinical Emotion and Cognition Research Laboratory, Goyang, Korea; 3 Department of Psychiatry, Ilsan Paik Hospital, Inje University, Goyang, Korea

## Abstract

This study aimed to examine the source-level cortical brain networks of post-traumatic stress disorder (PTSD) based on the graph theory using electroencephalography (EEG). Sixty-six cortical source signals were estimated from 78 PTSD and 58 healthy controls (HCs) of resting-state EEG. Four global indices (strength, clustering coefficient (CC), path length (PL) and efficiency) and one nodal index (CC) were evaluated in six frequency bands (delta, theta, alpha, low beta, high beta and gamma). PTSD showed decreased global strength, CC and efficiency, in delta, theta, and low beta band and enhanced PL in theta and low beta band. In low beta band, the strength and CC correlated positively with the anxiety scores, while PL had a negative correlation. In addition, nodal CCs were reduced in PTSD in delta, theta and low beta band. Nodal CCs of theta band correlated negatively with rumination and re-experience symptom scores; while, nodal CCs in low beta band correlated positively with anxiety and pain severity. Inefficiently altered and symptom-dependent changes in cortical networks were seen in PTSD. Our source-level cortical network indices might be promising biomarkers for evaluating PTSD.

## Introduction

Post-traumatic stress disorder (PTSD) is a unique mental illness with dysfunctional brain activities, demonstrated by symptoms such as re-experiences, avoidance and hyperarousal, as defined by the Diagnostic and Statistical Manual of Mental disorders (DSM-5).^1^ Abnormal function in specific regions such as amygdala, prefrontal cortex, anterior cingulate cortex, hippocampus and parahippocampus, known to be related to anxiety and anxiety-related memory, in individuals with PTSD were discovered using various neuroimaging tools.^2, 3, 4, 5, 6, 7^ Particularly, many studies have reported altered amygdala and frontal activation in PTSD.^8, 9^ Shin *et al.*^6^ have reported that PTSD patients showed amygdala hyperactivation and frontal hypoactivation, and found that these regional activities were significantly correlated with the Clinician-Administered PTSD Scale score.

However, small-scale region-based approaches, focusing only on specific brain regions, are limited in their capacity to promote understanding of complex human brain networks, because regions in the human brain are closely connected to allow efficient information processing. Hence, large-scale functional connectivity (FC) studies may better elucidate whole brain networks. Indeed, some functional magnetic resonance imaging (fMRI) studies have shown altered resting-state FC in some brain regions, including the amygdala, anterior cingulate cortex (ACC) and medial prefrontal cortex in patients with PTSD as compared to healthy controls (HCs).^10, 11, 12^

Electroencephalography (EEG) allows optimal observation of ongoing changes in brain activities, due to its high temporal resolution. Yet, few researchers have attempted to investigate FC in PTSD, using EEG to date, and have found some changes in FC in PTSD as compared to HCs.^13, 14, 15, 16, 17^ Cook *et al.*^13^ have demonstrated that PTSD patients with childhood trauma showed enhanced alpha and beta band coherence over the central and temporal areas, while Imperatori *et al.*^15^ have found increased alpha band FCs between the precuneus and the right inferior parietal lobe in individuals with PTSD, as compared to HCs, using lagged phase synchronization.

Recently, an increased number of researchers have focused on complex network constructions of the human brain and quantified global and nodal level complex networks based on graph theory (see the Supplementary Information 2 for more detailed explanation of each index).^18^ Especially, complex brain network dynamics could address neural mechanism and brain pathology of psychiatric disorders, such as PTSD.^18, 19^ For example, we have previously investigated complex networks in PTSD, using three network indices based on graph theory, which has recently been introduced as a theory for constructing human brain networks. We found reduced frontal nodal connection strength and communication efficiency in beta and gamma frequency bands in PTSD, as compared to HCs, and found that network values were significantly correlated with PTSD symptom scales. However, our previous study only focused on sensor-level (electrode-level) networks, leaving some concerns about spurious connections arising from volume conduction effects; moreover, regional brain characteristics could not be estimated. These shortcomings could be overcome by source-level network analysis.^17^

This study investigated brain cortical networks in PTSD using a source-level weighted network analysis of resting-state EEG. This allowed evaluation of regional cortical networks within a whole-brain framework. Furthermore, the relationships between psychiatric symptoms scales and the index of PTSD cortical network could be evaluated.

Among various psychiatric symptoms scales, we particularly focused on Impact of Event Scale-Revised (IES-R). IES-R is commonly used self-report scales for evaluating symptoms of PTSD,^20, 21^ and the relationships between IES-R and sensor-level EEG measurements such as several event-related potential (ERP) components^22, 23^ have been studied continuously. However, not yet, the relationships between source-level brain network and IES-R is uncovered. We explored that patients with PTSD would show an altered cortical brain network at both global and nodal levels, and that altered cortical network indices would significantly correlate with psychiatric symptom scores including IES-R.

## Materials and methods

### Participants

All subjects provided written informed consent, and the study protocol was approved by the Institutional Review Board of Inje University Ilsan Paik Hospital (2015-07-025).

Seventy-eight patients with PTSD (29 males and 49 females) and 58 HCs (30 males and 28 females) were recruited from the Psychiatry Department of the Inje University Ilsan Paik Hospital, Goyang, Korea, for this study. The diagnosis of PTSD was based on the Structured Clinical Interview for DSM-IV (SCID) Axis I Psychiatric Disorders^24^ by a psychiatrist. All patients were evaluated using brain computed tomography or MRI to rule out possible brain organicity. Patients were excluded if they had any abnormal brain imaging findings, a history of a loss of consciousness, and any memory loss about PTSD-causing traumatic events. HCs were recruited from the local community through flier and posters, and they were evaluated using the SCID Axis I Psychiatric Disorders^24^ and underwent a physical examination by a psychiatrist. HCs had no history of major trauma, such as a serious car accident, combat experience, sexual assault, serious physical injury and so on, and were not taking medications with potentially psychoactive effects.

The symptom evaluating scales used were as follows: subjective scales—IES-R (Cronbach *α*=0.93),^21, 25^ Beck Anxiety Inventory (BAI), Cronbach *α*=0.92),^26, 27^ State−Trait Anxiety Inventory (STAI-state and trait, Cronbach *α*=0.97),^27, 28^ Beck Depression Inventory (BDI, Cronbach *α*=0.89),^29, 30^ Insomnia Severity Index (ISI, Cronbach *α*=0.92),^31, 32^ Pain Anxiety Symptoms Scale (PASS, Cronbach *α*=0.91)^33, 34^ and Suicidal Ideation Questionnaire (SIQ, Cronbach *α*=0.97);^35, 36^ objective scales—the Hamilton Anxiety Rating Scale (HAM-A, Cronbach *α*=0.84)^37, 38^ and, ^27, 28^ Hamilton Depression Rating Scale (HAM-D, Cronbach *α*=0.79).^39, 40^ Also, all patients were currently receiving medications: selective serotonin reuptake inhibitors (*N*=67), venlafaxine (*N*=10), aripiprazole (*N*=5), quetiapine (*N*=17), lorazepam (*N*=37), clonazepam (*N*=27), diazepam (*N*=15) and alprazolam (*N*=35).

### EEG recordings and pre-processing

EEG signals were recorded using a NeuroScan SynAmps2 amplifier (Compumedics USA, El Paso, TX, USA) from 62 Ag/AgCl scalp electrodes that were evenly mounted on a QuickCap according to the extended international 10–20 system. Electrode impedances were <5 kΩ. The ground electrode was placed on the forehead and the reference electrodes were attached at the Cz electrode. The vertical electrooculogram channels were located above and below the right eye and the horizontal electrooculogram channels were placed on the outer canthus of each eye; EEG data were recorded with a 1−100-Hz band-pass filter at a sampling rate of 1000 Hz, with 60 Hz noise removed using a notch filter.

Resting-state EEGs were recorded for 5 min with the eyes closed. Gross artifacts such as muscle artifact were rejected by visual inspection by a skilled expert. Eye related artifacts were corrected using the standard correction algorithms implemented in the preprocessing software.^41^ The data were band-pass filtered at 1–55 Hz. The selection of the length of EEG data for source signal analysis was guided by a previous study, which reported that EEG data length of 40 s of showed acceptable reliability when analyzing resting-state conditions.^42^ The study showed that the reliability of EEG measurements increased with epoch lengths up to 40 s; longer epochs gave only marginal improvements. Thus, we selected 40.69 s of continuous EEG data, which did not contain significant physiological artifacts (amplitude exceeding±75 μV) at any site over all electrodes.^17, 43, 44^ Then, the data were segmented into 10 artifact-free epochs with a duration of 4.096 s per each epoch to efficiently estimate source signals.^45, 46, 47^

### Source localization

To estimate a time-series of source activities, the minimum-norm estimation was used, which was implemented in the eConnectome toolbox (Biomedical Functional Imaging and Neuroengineering Laboratory, University of Minnesota, Minneapolis, MN, USA).^48^ A three-layer boundary element method model, constructed from the MNI 152 standard template, was used to compute the lead field matrix. Cortical current density values at 7850 cortical vertices were evaluated for every time-point of each epoch. After estimating the cortical current density at every time-point, 66 nodes were selected from among the original cortical vertices. In our previous studies, we selected 314 nodes as evenly as possible;^17^ however, we found that these were too numerous to allow efficient estimation of brain regions. Thus, here, we evenly selected 66 nodes (33 per hemisphere) that were sampled based on the Brodmann areas (BA), excluding areas located deep in the brain (Supplementary Information). The representative value of each node was evaluated by averaging the cortical sources located in each node. A time-series of the cortical sources at each of the 66 nodes were band-pass filtered and divided into six frequency bands (delta (1–4 Hz), theta (4–8 Hz), alpha (8–12 Hz), low beta (12–22 Hz), high beta (22–30 Hz) and gamma (30–55 Hz)).

### Connectivity and network analysis

The FC between each pair of nodes was evaluated using phase-locking values (PLVs). PLVs were used as the measure of synchronization, because PLVs range from 0 to 1, and thus can be directly used to represent the connection strength in a weighted network analysis, without any further modification.

The weighted network was quantitatively analyzed based on graph theory.^18, 49, 50^ Four different global level weighted network indices were evaluated as follows: (1) Strength: the degree of connection strength in the network. The value of strength is estimated by sum of weights of links connected to the brain regions. (2) Clustering coefficient (CC): the degree which a node is clustered with neighbor’s nodes, and CC is calculated for the whole network. (3) Path length (PL): the summation of lengths between two nodes in whole network, and PL means overall connectedness of the whole network. (4) Efficiency: efficiency of information processing in the brain. In addition, the weighted nodal CC was evaluated for each node.

### Statistical analysis

The differences in cortical network characteristics at the global level between PTSD and HCs were investigated for each frequency band by using independent *t*-tests. At the nodal level, group differences were assessed using independent *t*-tests. At both global and nodal level, the *P*-value was adjusted using false discovery rate correction to avoid confounding by multiple testing. When significant differences were found in the network indices between the two groups, the effect-size (eta squared, *η*^2^) was calculated and significantly different nodes were defined with a 0.06-threshold (medium effect).^51^ Correlation analyses were performed to investigate the relationships between the network index and the symptom severity scores in PTSD, using Spearman’s correlation method with 1000 bootstrap replications to avoid multiple testing issues.

## Results

### Demographic data

Demographic and psychological characteristics of participants are demonstrated in Table 1. Patients with PTSD and HCs did not differ significantly in terms of sex (*P*=0.082), age (*P*=0.646) or education (*P*=0.120).

### Global level differences in cortical functional networks

The global level indices of strength, CC, PL and efficiency showed significant differences between PTSD and HCs (Table 2). Strength, CC, and efficiency were significantly decreased in PTSD patients as compared to HCs in the delta, theta and low beta. PL was significantly longer in PTSD patients than in HCs in the theta and low beta bands. To verify whether statistical results were affected by confound factor or not, we performed additional statistical task between PTSD patients (*n*=35) and PTSD patients with comorbidity (*n*=42). There was no significant results in global network measurements between PTSD patients and PTSD patients with comorbidity (*P*=NS) in delta, theta and low beta band.

### Nodal level differences in cortical functional networks

**Figure 1:**
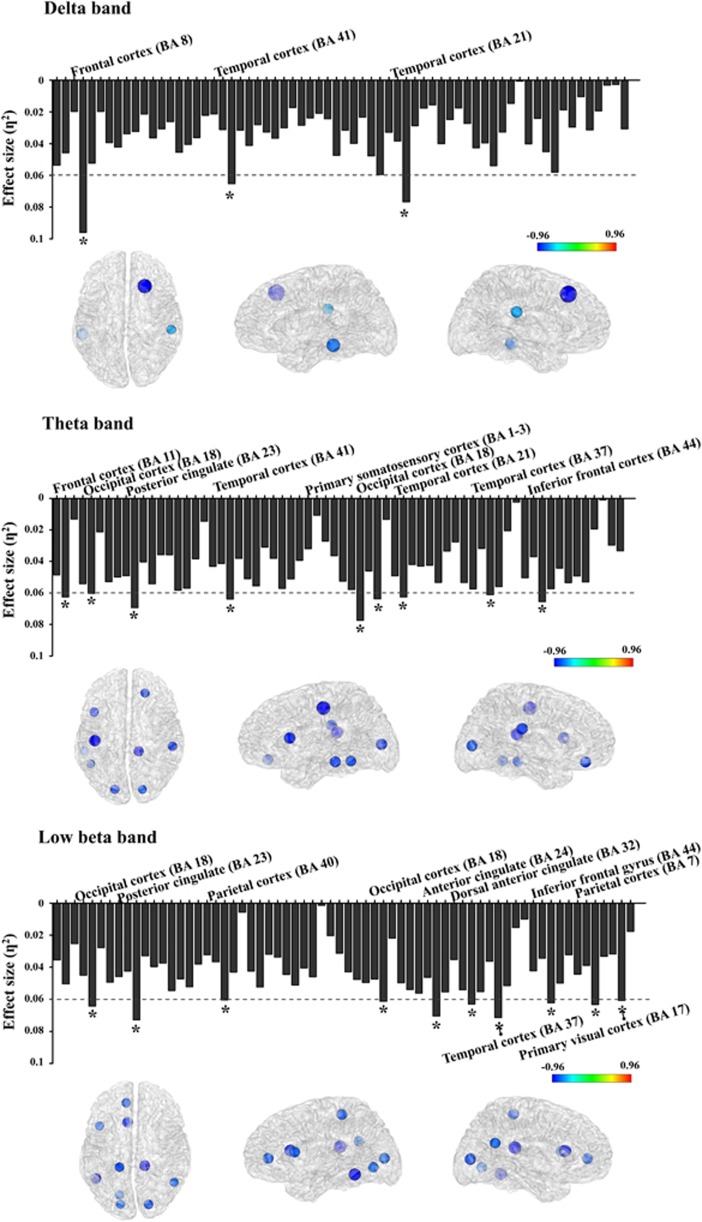
Effect-size of differences of nodal clustering coefficients between post-traumatic stress disorder (PTSD) and healthy controls (HCs) in three frequency bands. The threshold value was set as 0.06 (medium effect). In the brain model, the density of colors and size of circles represent the difference direction and effect-size, respectively. *η^2^>0.06.

For nodal level networks, the CC was investigated in the delta, theta and low beta bands, as these three frequency bands showed significant differences in global level networks. The nodal CC of PTSD patients was significantly decreased, virtually as whole nodes (*P*<0.05, false discovery rate corrected) in all three bands. Where significant differences were found, the effect-size exceeded 0.06 in each frequency band for the brain region; these were as follows: delta band—frontal (BA8) and temporal (BA21 and BA41) cortex; theta band—frontal (BA11 and BA44), occipital (BA8), posterior cingulate cortex (BA23; PCC), temporal (BA21, BA38, and BA41), and primary somatosensory cortex (BA1−3); low beta band—frontal (BA44), anterior cingulate (BA24 and BA32), parietal (BA7 and BA40), temporal (BA37) and occipital cortex (BA17 and BA18). Figure 1 illustrates the effect-size of differences in each nodal CC, as compared to that of HCs, for PTSD patients.

### Relationships between network indices and psychiatric scores

Relationships between global level indices and psychiatric scores were significant only in the low beta band. Here, PL correlated negatively with HAM-A (rho=−0.244, *P*=0.037) and STAI-state (rho=−0.256, *P*=0.047), while strength and CC were positively correlated with STAI-state (rho=0.257, *P*=0.046; rho=0.264, *P*=0.040, respectively).

**Figure 2:**
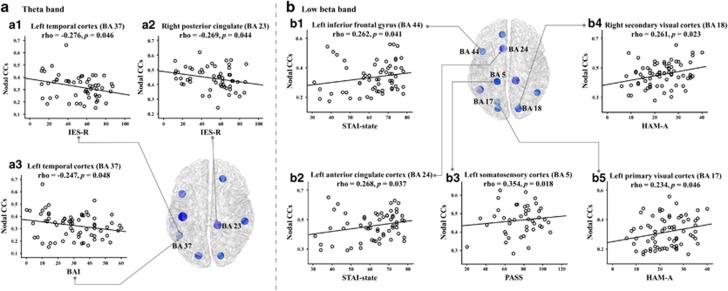
The relationships between nodal clustering coefficients (CCs) in the theta and low beta bands and psychiatric symptoms (refer to Table 4).

In the nodal level analysis, the relationships between nodal level indices and IES-R significant or not were represented in Table 3. The significant relationships between nodal level indices and psychiatric scores were found in the theta and low beta bands. In the theta band, the nodal CC located in the right PCC (BA23) correlated negatively with IES-R (rho=−0.269, *P*=0.044, Figure 2; a2); similarly, that in the left temporal cortex (BA37) correlated negatively with IES-R (rho=−0.276, *P*=0.046, Figure 2; a1) and BAI (rho=−0.247, *P*=0.048, Figure 2; a3), respectively. In the low beta band, the nodal CCs located in the left inferior frontal cortex (BA44) and left ACC (BA24) were positively correlated with STAI-state (rho=0.262, *P*=0.041, Figure 2; b1; rho=0.268, *P*=0.037, Figure 2; b2, respectively), while those in the right secondary visual cortex (BA18) and left primary visual cortex (BA17) were positively correlated with HAM-A (rho=0.261, *P*=0.023, Figure 2; b4; rho=0.234, *P*=0.046, Figure 2; b5), and that in the left somatosensory association cortex (BA5) correlated positively with PASS (rho=0.354, *P*=0.018, Figure 2; b3).

## Discussion

We have identified dysfunctional characteristics of cortical networks in patients with PTSD by using resting-state EEG. The global level indices of strength, CC, and efficiency were significantly decreased in PTSD as compared to HCs in the delta, theta, and low beta bands, while PL was enhanced in the theta and low beta band. Nodal level CCs were significantly reduced in PTSD patients as compared to HCs in the delta, theta and low beta bands. The nodal CCs of the theta band were negatively correlated with symptom scores (IES-R and BAI), which reflected rumination and re-experience symptoms; while the nodal CCs of the low beta band correlated positively with symptom scores (SAIT, HAMA and PASS), which reflect anxiety and pain symptoms.

### Global level network

PTSD patients showed significantly decreased strength, CC and efficiency, while PL was significantly prolonged as compared to HCs. In addition, in the low beta frequency band, strength and CC significantly positively correlated with STAI-state, and PL correlated negatively with HAM-A and STAI-state.

Decreased CC and increased PL could imply the disrupted clustering and inefficiently increased connectedness of brain networks in PTSD, respectively. Furthermore, decreased strength could imply weaker connection strength (looser links) compared to that of HCs in the brain networks of PTSD patients. Previously, networks in PTSD have been investigated using global network values; however, the results were inconsistent.^52, 53, 54, 55^ Jung *et al.*^55^ found that global network values were significantly reduced in individuals with partial PTSD symptoms as compared to HCs, based on fMRI findings. In addition, Lee *et al.*^17^ showed a decreased nodal degree and efficiency in PTSD patients in their EEG-based study. By contrast, Lei *et al.*^53^ have reported increased CC and decreased PL in patients with PTSD.

We found the relationships between altered networks and anxiety-related symptom scores in the low beta band. This band is traditionally related to anxiety symptoms and anxiety disorders.^56, 57^ Even though patients with PTSD showed increased overall connectedness and decreased clustering and connection strength as compared to HCs, their anxiety symptoms, *per se*, were closely related to the decreased overall connectedness, and increased local clustering and connection strength.

### Nodal level network: theta band

We found significantly decreased nodal CCs in the frontal, temporal, PCC, and visual cortex in PTSD. In addition, the nodal CCs of the left PCC were negatively correlated with IES-R, and that of the temporal cortex (BA37) were negatively correlated with IES-R and BAI.

IES-R items are known to be related to re-experience and rumination symptoms.^58, 59^ The re-experience of traumatic memory and rumination are crucial factors for understanding the re-experiencing characteristics of PTSD.^60^ Bennett and Wells^61^ found significant relationships between negative memory and rumination, and between rumination and IES symptoms, respectively, even if there was no direct relationship between negative memory and IES symptoms. According to fMRI studies, negative memory was related to the hippocampus, frontal cortex, PCC and ACC, which are regions that are also involved with rumination.^62^ In addition, Piguet *et al.*^63^ found that the visual cortex and insula were significantly related with the ruminative response scale. Ruminative tendencies have been reported to be associated with temporal lobe activity.^64^ The abnormal activity of these brain regions have been revealed in several anxiety disorders, including PTSD.^65, 66^ Moreover, it has been shown that theta oscillations are related to negative memory and rumination processes. Theta oscillations arise via the amygdala−hippocampal pathway during fear memory conditions,^67^ and theta band connectivity has been associated with personal rumination as opposed to nominal rumination.^68^

Our results of a disrupted brain network involving the theta band and significant relationships thereof with IES-R scores provides insight into rumination and re-experience symptoms in PTSD. More specifically, as the severity of rumination and re-experience symptoms increase, the functional clustering of left PCC and temporal nodes reduce. Overall, this suggested that disrupted theta band networks in the frontotemporal area reflect the pathology of PTSD, including abnormal memory, re-experience, and rumination.

### Nodal level network: low beta band

The nodal CCs of the low beta band were significantly decreased in several brain regions. Furthermore, anxiety symptom scores showed significant positive correlations with nodal CCs; more specifically, with the HAM-A in the right secondary visual cortex and left primary visual cortex, with the STAI-state in the left ACC and left inferior frontal cortex, and with the PASS in the left somatosensory association cortex.

Altered beta band activities have been demonstrated in previous studies of patients with PTSD. Begic *et al.*^69^ and Jokic-begic and Begic^70^ have reported that combat veterans with PTSD had increased beta activities in central regions compared to HCs. Increased beta power is known to occur during sleep in individuals who had undergone childhood maltreatment.^71^ Thus, disrupted beta band activity was assumed to be related to anxiety symptoms in patients with PTSD. Moreover, the regions in which we found abnormal cortical networks and significant symptomatic correlations have been areas of focus in previous PTSD studies. Hamner *et al.*^72^ argued that the ACC was an important brain region for understanding the anxiety symptoms of patients with PTSD. Lanius *et al.*^73^ have reported that reduced activity in the ACC and frontal cortex are associated with altered emotional modulation. In addition, Yin *et al.*^2^ found reduced brain activation in the insula, visual cortex and cerebellum during resting, and contended that altered activities in the visual area was related to processing of visual imagery in patients with PTSD. In line with these previous results, our findings of dysfunctional beta band cortical networks can reflect the pathophysiology, and particularly the anxiety symptoms^74, 75^ of PTSD. Taken together, patients with PTSD seem to have reduced functional clustering as compared to HCs, and the increased anxiety symptoms seem to produce additional local brain networking (hyperclustering around certain brain regions) in the ACC, visual cortex and inferior frontal cortex in patients with PTSD.

In addition, in PTSD patients, the nodal CCs of the somatosensory association cortex correlated significantly with the PASS score, reflecting anxiety and pain symptoms. Therefore, as the pain score increased, additional local brain networking was seen (hyperclustering around certain brain regions). Davis^76^ investigated the neural circuitry of pain, and revealed that the primary and secondary somatosensory cortex, thalamus, and ACC were involved in pain processing. Furthermore, Geuze *et al.*^77^ found that PTSD patients showed altered activities in the ACC, amygdala and somatosensory cortex during pain processing as compared to those in HCs. Thus, disrupted nodal CCs in the low beta band appear to be a critical aspect of the pathophysiology of anxiety and pain in patients with PTSD.

### Nodal level network: delta band

Moreover, patients with PTSD showed significantly decreased nodal CCs in the frontal and temporal area in the delta frequency band. Some researchers have reported diminished delta sleep responses in PTSD patients; this is intimately associated with hypothalamic corticotropin-releasing factor.^78, 79^ More studies are needed to quantify the altered brain networks of PTSD patients in the delta frequency band. A lot of researchers have already investigated the meanings of network indices and frequency bands. However, no study has yet integrated four network indices and six frequency bands, analyzing these variables as a whole. Thus, our present study is fairly exploratory, however, promising at the same time.

### Limitations and future works

According to several previous studies, PTSD patients showed decreased FCs between the default mode network (DMN) areas including the PCC, ACC, medial frontal and medial temporal regions during resting state. Also, DMN connectivity was significantly associated with symptom scores of PTSD. These results reflect the pathophysiology of PTSD patients such as self-related awareness, stimulus processing and memory. At nodal level network in present study, significantly reduced nodal CCs were appeared in PCC, ACC, frontal and temporal cortex, and these brain regions were in line with DMN. However, our results did not focused on DMN areas but that exploring whole brain networks. Therefore, in order to explore the exact DMN connectivity or network measures, further study is necessary focusing on specific region of interests are directly associated with DMN. On top of this, there are some limitations to the present study. All of the patients were on medication, and thus we could not control for possible confounding effects of the psychotropic medication. In addition, we did not use individual head models for EEG source imaging.

## Conclusion

Our results have demonstrated dysfunctional cortical networks at both global and nodal levels in PTSD patients, using resting-state EEG, and also found significant correlations between cortical network states and symptom severity scores. Rumination and re-experience symptoms are related to decreased theta band networks predominantly in the frontotemporal region. Anxiety and psychosomatic pain symptoms are related to altered low beta band networks, and hyper-clustering occurred with increased anxiety and pain symptoms. Our source-level cortical network indices provide insights into brain mechanisms of PTSD patients and may be promising biomarkers for evaluating PTSD patients.

## Figures and Tables

**Table 1:** Demographic data of post-traumatic stress disorder and healthy controls

	*PTSD*	*HCs*	P-*value*
Cases (*N*)	77	58	
Gender (male/female)	28/49	30/28	0.082^a^
Age (years)	40.92±11.93	39.98±11.63	0.646
Education	13.51±2.80	14.45±3.37	0.120
			
*Comorbidity (*N*, (%))*			
Major depressive disorder	32 (41.56)		
Obsessive compulsive disorder	3 (3.90)		
Generalized anxiety disorder	5 (6.49)		
Panic disorder	15 (19.48)		
			
*Symptom score*			
HAM-A	22.77±8.00		
HAM-D	23.24±9.49		
BDI	26.99±13.13		
BAI	29.48±15.44		
STAI-state	62.15±12.70		
STAI-trait	54.44±17.23		
IES-R	51.34±21.71		
ISI	18.11±5.94		
PASS	76.07±19.53		
SIQ	45.37±41.12		

Abbreviations: BAI, Beck Anxiety Inventory; BDI, Beck Depression Inventory; HAM-A, Hamilton Anxiety Rating Scale; HAM-D, Hamilton Depression Scale; HCs, healthy controls; IER-S, Impact of Event Scale-Revises; ISI, Insomnia Severity Index; PASS, Pain Anxiety Symptoms Scale; PTSD, post-traumatic stress disorder; SIQ, Suicidal Ideation Questionnaire; STAI, State-Trait Anxiety Inventory.

a Calculated by using *χ*^2^-test.

**Table 2:** Mean and s.d. values of global network indices of strength, clustering coefficient, path length and efficiency in six frequency bands

	*PTSD*	*HCs*	P*-value*
*Delta band*			
Strength	27.86±4.01	29.33±5.88	0.035*
CC	0.39±0.06	0.41±0.09	0.028*
PL	2.69±0.28	2.57±0.40	0.083
Efficiency	0.45±0.06	0.48±0.08	0.043*
			
*Theta band*			
Strength	29.06±4.39	31.50±5.40	0.025*
CC	0.42±0.07	0.45±0.09	0.019*
PC	2.67± 0.35	2.51±0.41	0.039*
Efficiency	0.47± 0.06	0.50±0.08	0.025*
			
*Alpha band*			
Strength	37.41±7.59	39.45±7.81	0.129
CC	0.54±0.12	0.58±0.13	0.125
PC	2.11±0.48	1.99±0.45	0.146
Efficiency	0.59±0.11	0.42±0.11	0.139
			
*Low beta band*			
Strength	26.70±4.01	29.33±5.88	0.025*
CC	0.39±0.06	0.41±0.09	0.019*
PL	2.69±0.28	2.57±0.40	0.042*
Efficiency	0.45±0.06	0.48±0.08	0.034*
			
*High beta band*			
Strength	23.18±4.26	24.53±4.86	0.129
CC	0.32±0.07	0.34±0.08	0.125
PL	2.95±0.41	3.15±0.46	0.161
Efficiency	0.39±0.06	0.41±0.07	0.139
			
*Gamma band*			
Strength	17.31±3.31	19.32±4.22	0.129
CC	0.25±0.05	0.26±0.06	0.125
PL	3.92±0.43	3.84±0.57	0.353
Efficiency	0.32±0.04	0.34±0.06	0.140

Abbreviations: CC, clustering coefficient; HCs, healthy controls; PL, path length; PTSD, post-traumatic stress disorder.

*P<0.05.

**Table 3:** The relationships between nodal level clustering coefficients and IES-R in delta, theta and low beta bands using Spearman’s method, with 1000 bootstrap replications

*Hemisphere*	*Structure*	*Brodmann area*	*rho*	P*-value*
*Delta band*				
	Frontal cortex	BA 8 (R)	−0.138	0.280
	Temporal cortex	BA 42 (R)	0.083	0.547
	Temporal cortex	BA 21 (L)	0.131	0.340
				
*Theta band*				
	Frontal cortex	BA 11 (R)	−0.073	0.937
		BA 44 (L)	−0.183	0.180
	Secondary visual cortex	BA 18 (R)	−0.062	0.651
		BA 18 (L)	−0.203	0.137
	**Posterior cingulate cortex**	**BA 23 (R)**	−**0.269**	0.044*
	Primary somatosensory cortex	BA 1-3 (L)	0.011	0.937
	**Temporal cortex**	BA 41 (R)	0.069	0.487
		BA 21 (L)	−0.104	0.452
		**BA 37 (L)**	−**0.276**	0.046*
				
*Low beta band*				
	Anterior cingulate cortex	BA 24 (L)	0.027	0.847
	Anterior cingulate cortex	BA 32 (L)	0.058	0.672
	Inferior frontal gyrus	BA 44 (L)	0.088	0.523
	Posterior cingulate cortex	BA 23 (R)	0.022	0.871
	Primary visual cortex	BA 17 (L)	−0.027	0.844
	Secondary visual cortex	BA 18 (R)	0.053	0.699
		BA 18 (L)	−0.047	0.733
	Somatosensory association cortex	BA 5 (L)	0.191	0.162
	Temporal cortex	BA 41 (R)	0.093	0.502
	Temporal pole	BA 39 (L)	0.056	0.685

Abbreviations: BA, Brodmann area; IES-R, Impact of Event Scale-Revises; L, left; R, right.

*P<0.05.

The bold letters represent significant correlation results.

**Table 4:** The significant relationships between nodal level clustering coefficients and symptom scores excluding Impact of Event Scale-Revises in theta and low beta bands using Spearman’s method, with 1000 bootstrap replications

*Psychiatric score*	*Structure*	*Brodmann area*	*rho*	P*-value*
*Theta band*				
BAI	Temporal cortex	BA 37 (L)	−0.247	0.048*
				
*Low beta band*				
HAM-A	Secondary visual cortex	BA 18 (R)	0.261	0.023*
	Primary visual cortex	BA 17 (L)	0.234	0.046*
STAI-state	Anterior cingulate cortex	BA 32 (L)	0.268	0.037*
	Inferior frontal gyrus	BA 44 (L)	0.262	0.041*
PASS	Somatosensory association cortex	BA 5 (L)	0.354	0.018*

Abbreviations: BAI, Beck Anxiety Inventory; HAM-A, Hamilton Anxiety Rating Scale; L, left; PASS, Pain Anxiety Symptoms Scale; R, right; STAI, State-Trait Anxiety Inventory. *P<0.05.

## Supplementary Material

Supplementarty Information
