# Supplementary material for: Disrupted cortical brain network in post-traumatic stress disorder patients: a resting-state electroencephalographic study
Source: Transl Psychiatry. 2017 Sep 12;7(9):e1231–. doi: 10.1038/tp.2017.200 (PMC5639244; doi:10.1038/tp.2017.200)
Supplement: Supplementarty Information [file tp2017200x1.docx]

**Network indices**

The weighted network was quantitatively analyzed based on graph theory. Four different global-level weighted network indices were evaluated as follows:

1) Strength: the degree of connection strength in the network. The value of strength is estimated by sum of weights of links connected to the brain regions.

2) Clustering coefficient: how strongly each node is connected with its neighbors. Clustering coefficient quantified he degree of clustering of a node or nodes. Clustering coefficient $C$ in each node is defined by

$$C_{i}= \frac{1}{K_{i}\left( K_{i}-1 \right)}\sum_{j,k\in G, j, k\neq i} \left( w_{ij}\cdot w_{jk}\cdot w_{ki} \right)^{\frac{1}{3}}$$

where $K$ is the number of neighbors of a vertex and $w$ is weights of links connected brain regions. Global clustering coefficient is defined as the average of the local clustering coefficients of all the vertices by

$$\bar{C}=\frac{1}{n}\sum_{i=1}^{n} C_{i}$$

3) Path length: how well the network nodes are communicating with each other. Path length estimated overall connectedness of the whole network. Path length $L$ is defined by

$$L= \frac{1}{N\left( N-1 \right)}\sum_{i, j\in G, i\neq j} d_{ij}$$

where $N$ is number of nodes, d is the shortest distance between node $i$ and $j$, and $d_{ij}$ is defined by

$$d_{ij}=\min\left( \frac{1}{w_{ji}}+\cdots+\frac{1}{w_{hj}} \right)$$

4) Global efficiency: efficiency of information processing in the brain. Global efficiency is defined by

$$E_{global}=\frac{1}{N\left( N-1 \right)}\sum_{j\in G} \frac{1}{d_{ij}}$$

Additionally, the weighted nodal clustering coefficient was evaluated for each node.

Supplementary figure 1. Distribution of the 66 nodes based on Brodmann area extracted from the original surface model, which were used for the connectivity analyses. ‘F’, ‘L’, and ‘R’ represent front view, left view and right view, respectively.


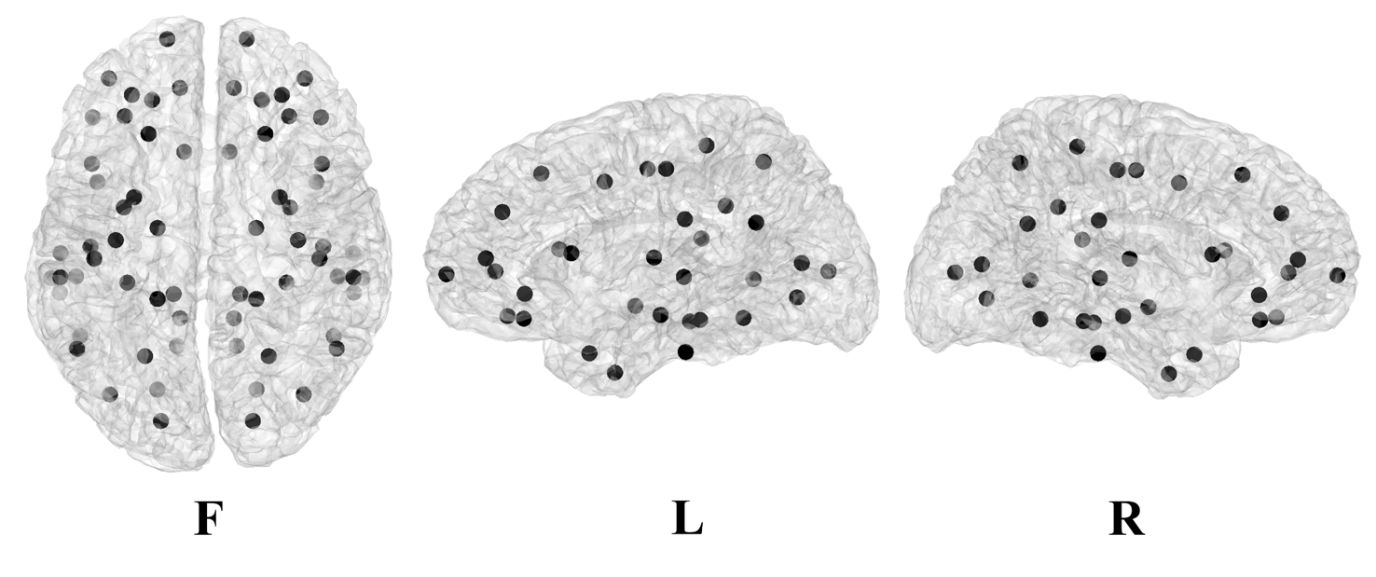


Supplementary table 1. The location of 66 nodes based on Brodmann area.

| **Number (L / R)** | **BA** |  | **Number (L / R)** | **BA** |  | **Number (L / R)** | **BA** |
| --- | --- | --- | --- | --- | --- | --- | --- |
| 1 / 34 | BA 10 |  | 12 / 45 | BA 26 – 29 |  | 23 / 56 | BA 43 |
| 2 / 35 | BA 11 |  | 13 / 46 | BA 30 |  | 24 / 57 | BA 44 |
| 3 / 36 | BA 1 – 3 |  | 14 / 47 | BA 31 |  | 25 / 58 | BA 45 |
| 4 / 37 | BA 8 |  | 15 / 48 | BA 32 |  | 26 / 59 | BA 46 |
| 5 / 38 | BA 18 |  | 16 / 49 | BA 34 |  | 27 / 60 | BA 47 |
| 6 / 39 | BA 19 |  | 17 / 50 | BA 35 |  | 28 / 61 | BA 4 |
| 7 / 40 | BA 20 |  | 18 / 51 | BA 37 |  | 29 / 62 | BA 5 |
| 8 / 41 | BA 21 |  | 19 / 52 | BA 38 |  | 30 / 63 | BA 6 |
| 9 / 42 | BA 22 |  | 20 / 53 | BA 39 |  | 31 / 64 | BA 7 |
| 10 / 43 | BA 23 |  | 21 / 54 | BA 40 |  | 32 / 65 | BA 17 |
| 11 / 44 | BA 24 |  | 22 / 55 | BA 41 |  | 33 / 66 | BA 9 |

BA: Brodmann area

Supplementary table 2. The relationships between global-level indices (strength, clustering coefficient, path length, and efficiency) and symptom scores in delta, theta, and low beta bands using Spearman’s method, with 1,000 bootstrap replications.

| **Structure** | HAM-A | | HAM-D | | BDI | | BAI | | STAI-state | | STAI-trait | | IES-R | | ISI | | PASS | | SIQ | |
| --- | --- | --- | --- | --- | --- | --- | --- | --- | --- | --- | --- | --- | --- | --- | --- | --- | --- | --- | --- | --- |
|  | rho | *p* | rho | *p* | rho | *p* | rho | *p* | rho | *p* | rho | *p* | rho | *p* | rho | *p* | rho | *p* | rho | *p* |
| **Delta band** |  |  |  |  |  |  |  |  |  |  |  |  |  |  |  |  |  |  |  |  |
| Strength | 0.102 | 0.393 | 0.046 | 0.700 | 0.047 | 0.699 | -0.058 | 0.642 | 0.056 | 0.667 | 0.026 | 0.843 | 0.093 | 0.497 | -0.076 | 0.590 | 0.076 | 0.622 | 0.155 | 0.277 |
| Clustering coefficient | 0.100 | 0.399 | 0.045 | 0.701 | 0.036 | 0.770 | -0.075 | 0.547 | 0.043 | 0.742 | 0.023 | 0.858 | 0.072 | 0.599 | -0.080 | 0.570 | 0.046 | 0.768 | 0.140 | 0.326 |
| Path length | -0.079 | 0.506 | -0.036 | 0.761 | -0.032 | 0.732 | 0.083 | 0.506 | -0.065 | 0.616 | -0.049 | 0.709 | -0.056 | 0.686 | 0.069 | 0.623 | -0.068 | 0.663 | -0.159 | 0.266 |
| Efficiency | 0.109 | 0.358 | 0.051 | 0.665 | 0.067 | 0.583 | -0.039 | 0.755 | 0.076 | 0.558 | 0.044 | 0.737 | 0.105 | 0.444 | -0.050 | 0.721 | 0.104 | 0.503 | 0.188 | 0.186 |
| **Theta band** |  |  |  |  |  |  |  |  |  |  |  |  |  |  |  |  |  |  |  |  |
| Strength | 0.099 | 0.407 | 0.011 | 0.929 | 0.067 | 0.583 | -0.128 | 0.300 | 0.133 | 0.308 | 0.199 | 0.124 | -0.084 | 0.542 | -0.026 | 0.853 | 0.093 | 0.549 | 0.003 | 0.983 |
| Clustering coefficient | 0.102 | 0.388 | 0.013 | 0.916 | 0.072 | 0.552 | -0.133 | 0.282 | 0.135 | 0.299 | 0.197 | 0.128 | -0.096 | 0.486 | -0.031 | 0.826 | 0.089 | 0.568 | -0.010 | 0.945 |
| Path length | -0.095 | 0.422 | 0.002 | 0.956 | -0.059 | 0.630 | 0.148 | 0.232 | -0.121 | 0.353 | -0.162 | 0.213 | 0.100 | 0.468 | 0.033 | 0.817 | -0.105 | 0.497 | 0.028 | 0.846 |
| Efficiency | 0.092 | 0.437 | 0.008 | 0.946 | 0.070 | 0.565 | -0.124 | 0.319 | 0.143 | 0.273 | 0.203 | 0.117 | -0.49 | 0.580 | -0.018 | 0.897 | 0.091 | 0.558 | 0.005 | 0.974 |
| **Low beta band** |  |  |  |  |  |  |  |  |  |  |  |  |  |  |  |  |  |  |  |  |
| Strength | 0.226 | 0.054 | 0.072 | 0.541 | 0.237 | 0.049 | 0.022 | 0.862 | **0.257** | **0.046** | 0.152 | 0.241 | 0.062 | 0.656 | 0.162 | 0.246 | 0.106 | 0.495 | 0.007 | 0.959 |
| Clustering coefficient | 0.226 | 0.054 | 0.079 | 0.506 | 0.232 | 0.053 | 0.019 | 0.877 | **0.264** | **0.040** | 0.148 | 0.254 | 0.055 | 0.691 | 0.153 | 0.274 | 0.106 | 0.494 | 0.003 | 0.984 |
| Path length | **-0.244** | **0.037** | -0.092 | 0.435 | -0.204 | 0.091 | -0.019 | 0.876 | **-0.256** | **0.047** | -0.117 | 0.369 | -0.057 | 0.682 | -0.154 | 0.270 | -0.151 | 0.329 | 0.005 | 0.972 |
| Efficiency | 0.225 | 0.056 | 0.066 | 0.574 | 0.234 | 0.052 | 0.024 | 0.850 | 0.251 | 0.051 | 0.151 | 0.247 | 0.065 | 0.619 | 0.167 | 0.232 | 0.098 | 0.526 | 0.014 | 0.922 |

Hamilton Anxiety Rating Scale: HAM-A, Hamilton Depression Rating Scale: HAM-D, Beck Depression Inventory: BDI, Beck Anxiety Inventory: BAI, State-Trait Anxiety Inventory: STAI, Impact of Event Scale-Revises: IER-S, Insomnia Severity Index: ISI, Pain Anxiety Symptoms Scale: PASS, Suicidal Ideation Questionnaire: SIQ

Supplementary table 3. The relationships between nodal-level clustering coefficients and symptom scores in delta, theta, and low beta bands using Spearman’s method, with 1,000 bootstrap replications.

**3-1. Delta frequency band**

| **Structure** | HAM-A | | HAM-D | | BDI | | BAI | | STAI-state | | STAI-trait | | IES-R | | ISI | | PASS | | SIQ | |
| --- | --- | --- | --- | --- | --- | --- | --- | --- | --- | --- | --- | --- | --- | --- | --- | --- | --- | --- | --- | --- |
|  | rho | *p* | rho | *p* | rho | *p* | rho | *p* | rho | *p* | rho | *p* | rho | *p* | rho | *p* | rho | *p* | rho | *p* |
| **Right** |  |  |  |  |  |  |  |  |  |  |  |  |  |  |  |  |  |  |  |  |
| Frontal  (BA 18) | -0.069 | 0.563 | -0.140 | 0.234 | 0.076 | 0.535 | 0.000 | 0.999 | 0.036 | 0.781 | -0.010 | 0.937 | -0.148 | 0.280 | -0.059 | 0.677 | -0.006 | 0.969 | 0.175 | 0.220 |
| Temporal  (BA 41) | 0.022 | 0.856 | -0.028 | 0.811 | 0.042 | 0.734 | 0.028 | 0.822 | 0.097 | 0.459 | 0.126 | 0.335 | 0.083 | 0.547 | 0.065 | 0.643 | -0.217 | 0.157 | 0.035 | 0.810 |
| **Left** |  |  |  |  |  |  |  |  |  |  |  |  |  |  |  |  |  |  |  |  |
| Temporal  (BA 21, R) | -0.039 | 0.748 | -0.098 | 0.406 | 0.063 | 0.605 | -0.027 | 0.832 | 0.030 | 0.817 | 0.128 | 0.325 | 0.131 | 0.340 | 0.155 | 0.269 | 0.182 | 0.237 | 0.190 | 0.181 |

Hamilton Anxiety Rating Scale: HAM-A, Hamilton Depression Rating Scale: HAM-D, Beck Depression Inventory: BDI, Beck Anxiety Inventory: BAI, State-Trait Anxiety Inventory: STAI, Impact of Event Scale-Revises: IER-S, Insomnia Severity Index: ISI, Pain Anxiety Symptoms Scale: PASS, Suicidal Ideation Questionnaire: SIQ

**3-2. Theta frequency band**

| **Structure** | HAM-A | | HAM-D | | BDI | | BAI | | STAI-state | | STAI-trait | | IES-R | | ISI | | PASS | | SIQ | |
| --- | --- | --- | --- | --- | --- | --- | --- | --- | --- | --- | --- | --- | --- | --- | --- | --- | --- | --- | --- | --- |
|  | rho | *p* | rho | *p* | rho | *p* | rho | *p* | rho | *p* | rho | *p* | rho | *p* | rho | *p* | rho | *p* | rho | *p* |
| **Right** |  |  |  |  |  |  |  |  |  |  |  |  |  |  |  |  |  |  |  |  |
| Frontal  (BA 11) | 0.122 | 0.303 | 0.030 | 0.800 | 0.082 | 0.500 | -0.112 | 0.368 | 0.164 | 0.206 | 0.216 | 0.095 | -0.074 | 0.593 | -0.009 | 0.948 | 0.107 | 0.491 | -0.002 | 0.991 |
| Occipital  (BA 18) | 0.132 | 0.265 | 0.040 | 0.736 | 0.073 | 0.549 | -0.126 | 0.310 | 0.100 | 0.444 | 0.250 | 0.113 | -0.062 | 0.651 | -0.041 | 0.770 | 0.097 | 0.532 | -0.032 | 0.825 |
| Posterior cingulate  (BA 23) | 0.010 | 0.936 | -0.082 | 0.489 | 0.024 | 0.846 | -0.216 | 0.079 | 0.073 | 0.577 | 0.060 | 0.645 | **-0.269** | **0.044** | -0.192 | 0.169 | 0.000 | 0.998 | -0.067 | 0.461 |
| Temporal  (BA 41) | 0.136 | 0.250 | -0.039 | 0.739 | 0.068 | 0.573 | -0.008 | 0.948 | 0.006 | 0.962 | 0.179 | 0.169 | 0.096 | 0.487 | 0.046 | 0.745 | -0.006 | 0.971 | -0.093 | 0.515 |
| **Left** |  |  |  |  |  |  |  |  |  |  |  |  |  |  |  |  |  |  |  |  |
| Primary somatosensory  (BA 1-3) | 0.082 | 0.488 | -0.041 | 0.730 | -0.032 | 0.791 | -0.040 | 0.747 | 0.175 | 0.177 | 0.101 | 0.438 | 0.011 | 0.937 | 0.093 | 0.510 | 0.240 | 0.116 | 0.000 | 0.999 |
| Occipital  (BA 18) | 0.061 | 0.607 | -0.027 | 0.817 | 0.021 | 0.861 | -0.101 | 0.415 | 0.036 | 0.781 | 0.152 | 0.242 | -0.203 | 0.137 | -0.104 | 0.460 | 0.041 | 0.789 | 0.048 | 0.740 |
| Temporal  (BA 21) | 0.060 | 0.615 | -0.004 | 0.974 | 0.110 | 0.367 | -0.145 | 0.243 | 0.134 | 0.302 | 0.162 | 0.212 | -0.104 | 0.452 | -0.027 | 0.848 | 0.257 | 0.092 | 0.050 | 0.726 |
| Temporal  (BA 37) | 0.118 | 0.322 | 0.047 | 0.693 | -0.001 | 0.993 | **-0.247** | **0.048** | 0.028 | 0.828 | 0.119 | 0.360 | **-0.276** | **0.046** | -0.089 | 0.526 | 0.105 | 0.499 | -0.177 | 0.213 |
| Inferior frontal  (BA 44) | 0.121 | 0.307 | 0.061 | 0.605 | 0.106 | 0.383 | -0.152 | 0.218 | 0.136 | 0.297 | 0.156 | 0.231 | -0.183 | 0.180 | -0.062 | 0.658 | 0.049 | 0.750 | 0.084 | 0.556 |

Hamilton Anxiety Rating Scale: HAM-A, Hamilton Depression Rating Scale: HAM-D, Beck Depression Inventory: BDI, Beck Anxiety Inventory: BAI, State-Trait Anxiety Inventory: STAI, Impact of Event Scale-Revises: IER-S, Insomnia Severity Index: ISI, Pain Anxiety Symptoms Scale: PASS, Suicidal Ideation Questionnaire: SIQ

**3-3. Low beta frequency band**

| **Structure** | HAM-A | | HAM-D | | BDI | | BAI | | STAI-state | | STAI-trait | | IES-R | | ISI | | PASS | | SIQ | |
| --- | --- | --- | --- | --- | --- | --- | --- | --- | --- | --- | --- | --- | --- | --- | --- | --- | --- | --- | --- | --- |
|  | rho | *p* | rho | *p* | rho | *p* | rho | *p* | rho | *p* | rho | *p* | rho | *p* | rho | *p* | rho | *p* | rho | *p* |
| **Right** |  |  |  |  |  |  |  |  |  |  |  |  |  |  |  |  |  |  |  |  |
| Occipital  (BA 11) | **0.261** | **0.023** | 0.111 | 0.348 | 0.199 | 0.099 | 0.005 | 0.968 | 0.199 | 0.125 | 0.138 | 0.291 | 0.053 | 0.699 | 0.145 | 0.299 | 0.073 | 0.638 | -0.019 | 0.896 |
| Posterior cingulate  (BA 23) | 0.197 | 0.095 | 0.053 | 0.657 | 0.215 | 0.074 | 0.003 | 0.980 | 0.252 | 0.050 | 0.167 | 0.199 | 0.022 | 0.871 | 0.120 | 0.397 | 0.062 | 0.689 | 0.021 | 0.886 |
| Parietal  (BA 40) | 0.227 | 0.050 | 0.072 | 0.540 | 0.160 | 0.185 | -0.034 | 0.786 | 0.219 | 0.091 | 0.174 | 0.180 | 0.093 | 0.502 | 0.090 | 0.523 | 0.123 | 0.428 | -0.001 | 0.994 |
| **Left** |  |  |  |  |  |  |  |  |  |  |  |  |  |  |  |  |  |  |  |  |
| Occipital  (BA 18) | 0.195 | 0.099 | 0.049 | 0.679 | 0.109 | 0.367 | -0.048 | 0.698 | 0.126 | 0.333 | 0.084 | 0.522 | -0.047 | 0.733 | 0.044 | 0.757 | 0.114 | 0.460 | -0.044 | 0.762 |
| Anterior cingulate  (BA 24) | 0.087 | 0.465 | -0.082 | 0.458 | 0.177 | 0.143 | 0.029 | 0.818 | 0.218 | 0.091 | 0.084 | 0.522 | 0.027 | 0.847 | 0.121 | 0.387 | 0.119 | 0.440 | 0.097 | 0.499 |
| Dorsal anterior cingulate  (BA32) | 0.224 | 0.057 | 0.054 | 0.650 | 0.217 | 0.071 | -0.005 | 0.969 | **0.268** | **0.037** | 0.136 | 0.297 | 0.058 | 0.672 | 0.137 | 0.330 | 0.129 | 0.405 | 0.039 | 0.784 |
| Temporal  (BA37) | 0.205 | 0.082 | 0.066 | 0.578 | 0.231 | 0.054 | 0.015 | 0.905 | 0.254 | 0.051 | 0.151 | 0.244 | 0.056 | 0.685 | 0.134 | 0.340 | 0.052 | 0.737 | 0.009 | 0.950 |
| Inferior frontal  (BA 44) | 0.139 | 0.241 | 0.033 | 0.780 | 0.167 | 0.167 | 0.045 | 0.718 | **0.262** | **0.041** | 0.155 | 0.234 | 0.088 | 0.523 | 0.186 | 0.182 | 0.043 | 0.783 | 0.131 | 0.360 |
| Somatosensory  (BA 5) | 0.229 | 0.051 | 0.092 | 0.437 | 0.097 | 0.424 | 0.173 | 0.162 | 0.176 | 0.176 | 0.027 | 0.834 | 0.191 | 0.162 | 0.077 | 0.584 | **0.354** | **0.018** | -0.158 | 0.270 |
| Primary visual  (BA 17) | **0.234** | **0.046** | 0.053 | 0.653 | 0.230 | 0.055 | 0.012 | 0.921 | 0.178 | 0.169 | 0.152 | 0.241 | -0.027 | 0.844 | 0.156 | 0.264 | 0.107 | 0.490 | -0.182 | 0.200 |

Hamilton Anxiety Rating Scale: HAM-A, Hamilton Depression Rating Scale: HAM-D, Beck Depression Inventory: BDI, Beck Anxiety Inventory: BAI, State-Trait Anxiety Inventory: STAI, Impact of Event Scale-Revises: IER-S, Insomnia Severity Index: ISI, Pain Anxiety Symptoms Scale: PASS, Suicidal Ideation Questionnaire: SIQ
